# MDM2- an indispensable player in tumorigenesis

**DOI:** 10.1007/s11033-023-08512-3

**Published:** 2023-06-14

**Authors:** Aasma Zafar, Muhammad Jawad Khan, Aisha Naeem

**Affiliations:** 1 Department of Biosciences, COMSATS University, Islamabad, 45550 Pakistan; 2 Department of Oncology, Lombardi Comprehensive Cancer Center, Georgetown University Medical Center, 20057 Washington, DC U.S.; 3 Qatar University Health, Qatar University, P.O. Box 2713, Doha, Qatar

**Keywords:** MDM2, TP53, Apoptosis, Cell signaling, Metabolism

## Abstract

Murine double minute 2 (MDM2) is a well-recognized molecule for its oncogenic potential. Since its identification, various cancer-promoting roles of MDM2 such as growth stimulation, sustained angiogenesis, metabolic reprogramming, apoptosis evasion, metastasis, and immunosuppression have been established. Alterations in the expression levels of MDM2 occur in multiple types of cancers resulting in uncontrolled proliferation. The cellular processes are modulated by MDM2 through transcription, post-translational modifications, protein degradation, binding to cofactors, and subcellular localization. In this review, we discuss the precise role of deregulated MDM2 levels in modulating cellular functions to promote cancer growth. Moreover, we also briefly discuss the role of MDM2 in inducing resistance against anti-cancerous therapies thus limiting the benefits of cancerous treatment.

.

## Introduction

The murine double minute 2 (*MDM2*) gene (also referred to as human double minute 2 (*HDM2*)) is well recognized for its growth-promoting role in various cancers [1]. The pathogenic role of *MDM2* in initiation, progression, metastasis, and chemotherapy resistance of cancer is majorly attributed to gene mutation and deregulated expression [2]. Genomic amplification and altered MDM2 levels are associated with unfavorable prognosis, poor response to chemotherapy and target therapy, and adverse clinicopathological parameters in many cancers [3–5].

The MDM2 reprograms many biological processes that support malignant transformations e.g., cell growth, angiogenesis, metabolism, apoptosis evasion, and metastasis [6]. The deregulated *MDM2* gene expression is ascribed to a variety of molecular and regulatory mechanisms. These processes include increased promoter strength caused by transversion of T to G at position 309 (SNP309) [7], increased transcription and translation of the gene[8], escalation in *MDM2* gene copy number[9] or dysfunctional *MDM2* regulators e.g., tumor protein p53 (TP53). The occurrence of splice variants of *MDM2* also contributes to the increased aggressiveness of various cancers [10]. Owing to its diverse functioning and huge significance in anti-apoptosis, various anti-cancer therapies targeting MDM2 have been developed [11]. This review aims to summarize the diversified cancer-promoting roles of *MDM2*.

## MDM2 empowers cancers cells to escape TP53-mediated cell death

MDM2 helps cancerous cells to evade death through a variety of mechanisms. Anti-apoptotic role of MDM2 is historically established after the discovery of the physical association of MDM2 with a tumor suppressor protein, TP53 [12]. The association of MDM2 and TP53 led to the formulation of the hypothesis that MDM2 acts as a negative regulator of TP53 [9]. Shortly after, the research provided pieces of evidence in support of the hypothesis reinforcing the antagonistic role of MDM2 for TP53 [12].

**Fig. 1:**
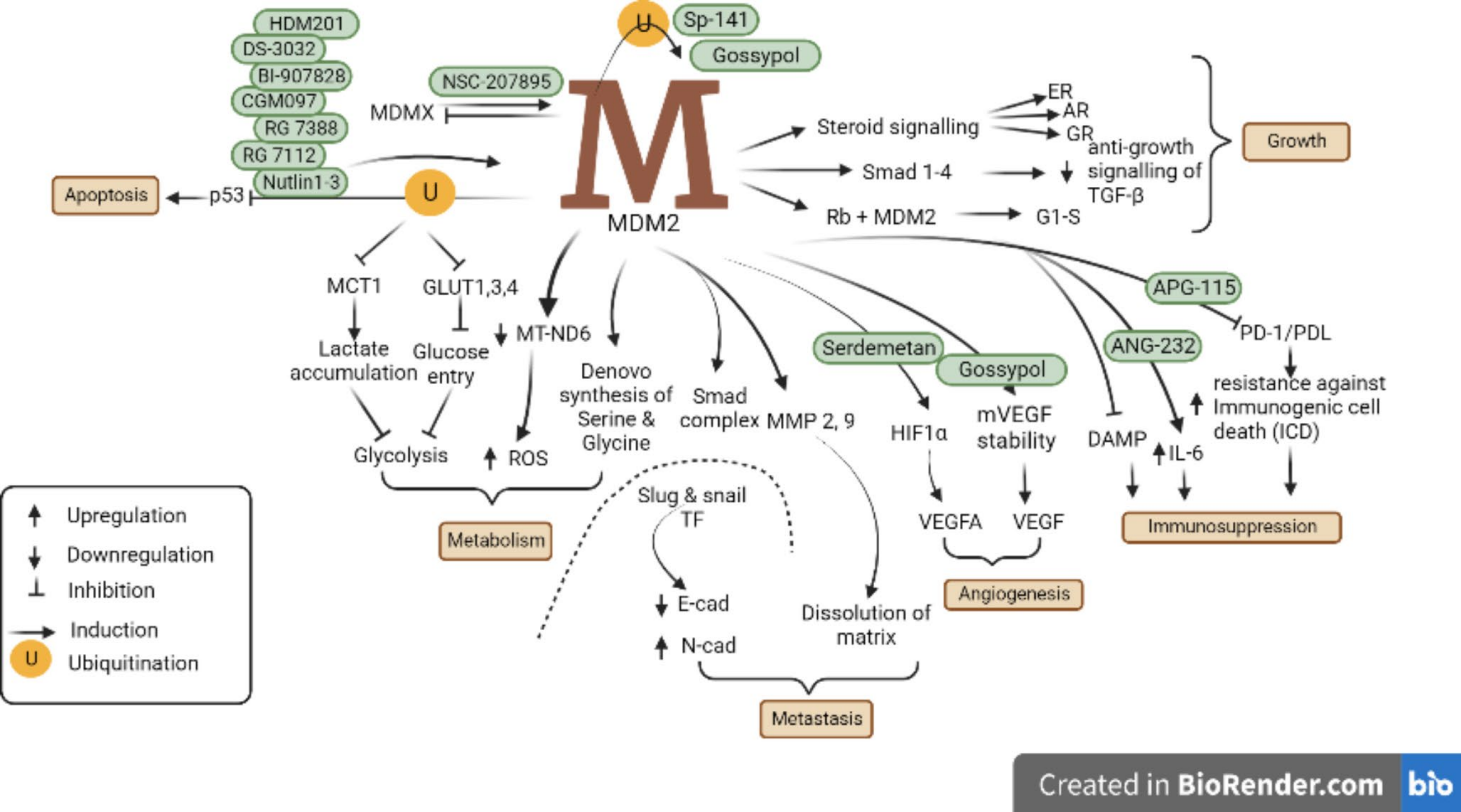
Multifaceted role of MDM2 in tumorigenesis. MDM2 regulates multiple processes of a cell including apoptosis, growth, angiogenesis, metabolism and metastasis. It also modulates the response of cancerous cells toward immunosuppressiveness. Multiple inhibitors have been synthesized to target MDM2-regulated pathways and induce apoptosis in cancerous cells. Abbreviation: MDM2, Murine Double Minute 2; MDMX, Murine Double Minute X; TP53, Tumor Protein p53; ER, Estrogen Receptor; AR, Androgen Receptor; GR, Glucocorticoid Receptors; Rb, Retinoblastoma; G1-S, G1 to S phase transition; TGF-β, Transforming growth Factor-beta; MCT1, Monocarboxylate Transporter 1; GLUT1, Glucose Transporter 1; GLUT3, Glucose Transporter 3; GLUT4, Glucose Transporter 4; MT-ND6, Mitochondrially encoded NADH Dehydrogenase 6; VEGF, Vascular Epithelial Growth Factors; VEGFA, Vascular Epithelial Growth Factor A; HIF1α, Hypoxia-Inducible Factor-alpha; IL-6, Interleukin 6; TF, Transcription factor; E-cad, E-cadherin; N-cad, N-cadherin; ROS, Reactive Oxidative Species; MMP-2, Matrix Metalloproteinase 2; MMP-9, Matrix Metalloproteinase 9; DAMP, Damage-Associated Molecular Pattern; PD-1/PDL, Programmed death-1/ programmed death ligand; Smad, Small mothers against decapentaplegic

*TP53* gene was first identified in 1979 as a partner of large T-antigen (inducers of tumors) of Simian Virus 40 (SV40) [13]. Several lines of historical and recent evidence suggest contradictory roles of TP53 in regulating cell fate [14–16]. The diverse functioning of TP53 as a tumor suppressor includes the regulation of expression of genes ensuing cell cycle arrest, senescence, and apoptosis in response to stress [17–19]. Paradoxically in many studies, high levels of TP53 protected cells from stress-induced death and led to chemo-resistant however knocking down TP53 levels was found counteractive [15]. The deregulated TP53 expression along with a high frequency of *TP53* mutations is associated with poor prognosis and enhanced chemoresistance in most, if not all, cancers[14]. TP53 activates MDM2 which in turn regulates the levels of TP53 in cells [20]. MDM2 variants deficient in the TP53 binding domain, are unable to inhibit TP53 leading to uncontrolled cellular proliferation [21]. Hence, the autoregulatory mechanism of TP53 through MDM2 maintains tight control of TP53 levels in cells and protects cells from the detrimental effects of high levels of TP53 on their growth and development [17] (Fig. 1).

In addition, MDM2 ensures the regulation of TP53 in cells through a variety of other mechanisms. These include driving TP53 out of the nucleus [22], preventing the interaction of TP53 with co-activators [23], and recruiting repressors to impede the transcription of *TP53* [24]. Furthermore, MDM2 ubiquitinates TP53 for its degradation by the proteasomal machinery of cells thus ensuring cell survival [25]. MDM2-mediated ubiquitination occurs exclusively in the nucleus[26] while proteasomal mortification can occur in the nucleus or cytosol as 26 S proteasomes exist in abundance at both sites [27]. In addition to ubiquitinating TP53, MDM2 inhibits its transcription by adding ubiquitin-like molecule Nedd8 (neural precursor cell expressed developmentally downregulated 8), a process known as neddylation [28]. A recent study demonstrated that phosphorylation of MDM2 on Y281 and Y302 switches its activity from ubiquitination to neddylation E3 ligase [29]. Ribosomal proteins (RPS27 and RPS27-like) are stabilized by MDM2-mediated neddylation, which improves the survival of tumorous cells [30].

## MDM2 promotes cell growth

**Fig. 2:**
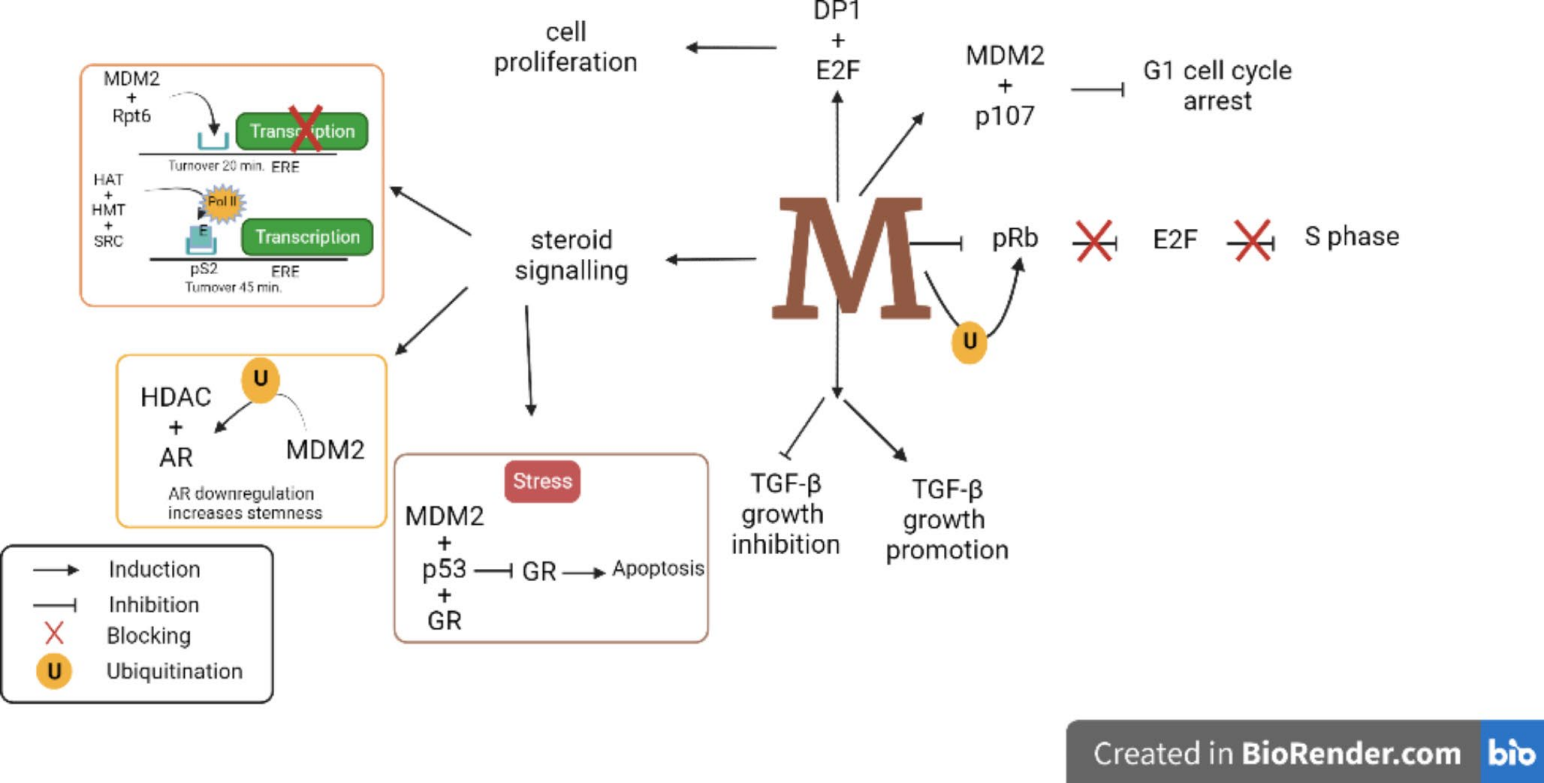
MDM2 regulation of cell growth. MDM2 regulates cell growth by blocking the inhibition of pRb on E2F allowing the progression to S phase. MDM2 interaction with p107 and DP1 and E2F complex allows cell proliferation. Through steroid signaling, MDM2 regulates the cell cycle by inhibiting AR, GR and ligand-free ER. MDM2 also brings about a shift in the role of TGF-β from growth inhibition to growth promotion. Abbreviations: MDM2, Murine Double Minute 2; DP1, Dimerization Partner 1; E2F, Elongation Factor 2; HDAC, Histone Deacetyltransferase; ER, Estrogen Receptor; AR, Androgen Receptor; GR, Glucocorticoid Receptors; Pol ll, polymerase ll; HAT, Histone Acetyltransferase; HMT, Histone Methyltransferase; SRC, Steroid Receptor Coactivator-1; TGF-β, Transforming Growth Factor-beta; pS2, Presenilin-2; ERE, Estrogen-Responsive Element

The cell growth-promoting activity of the MDM2 protein is regulated through reprogramming pathways and networks including TP53 [31], retinoblastoma (Rb) [32], transforming growth factor-beta (TGF-β) [33], steroid and androgen receptor (AR) [34] at various molecular levels [35, 36] (Fig. 2). Unrestrained cell proliferation is facilitated by MDM2 in TP53-dependent [2] as well as TP53-independent manner [37]. Through negative regulation of *TP53* expression, MDM2 helps cells to evade death signals, thus promoting the growth of tumorous tissue [2]. In a TP53-independent manner, MDM2 promotes cellular aging through negative regulation of genes involved in maintaining genomic stability e.g., Werner syndrome RecQ-like helicase (*WRN*). Initially, cellular aging was thought to delay the progression of cancer [38]. In contrast, recent findings support the notion that the secretory nature of senescent cells promotes the stimulation of tumor aggressiveness [39].

The impact of MDM2 on cell cycle progression through its interaction with retinoblastoma (Rb) family members is also well studied [40]. Rb family members, known as pocket proteins p105, p107, and p130 (Rb-like proteins), are involved in governing proliferation, differentiation, and apoptosis [41]. The pRb proteins inhibit the induction of the S-phase of the cell cycle by negatively regulating elongation factor 2 (E2F), an essential mediator of protein synthesis. Following ubiquitination, MDM2 degrades pRb thus releasing E2F from the inhibition of pRb [42]. The association of MDM2 with p107 in TP53 deficient cells has been shown to subdue G1 cell cycle arrest thus instigating cell cycle progression [43].

MDM2 also triggers cell proliferation by promoting the activation of a complex formed by E2F and DP1 (dimerization partner 1) [44]. E2F enhances the activation of Akt through the PI3K/Akt pathway [45]. It is also assumed that upregulated *MDM2* expression is responsible for shifting the balance toward cell survival by uplifting *Akt* through E2F and lowering *TP53* activity in cells [46]. Moreover, MDM2 attenuates the binding of E2F1 to DNA by misfolding E2F1 in the deterrence of E2F1-mediated induction of apoptosis [47].

*MDM2* also stimulates cell growth by redirecting the network of another multifunctional cytokine, transforming growth factor-beta (TGF-β) [48]. Like E2F, TGF-β also acts as a cell growth promoter or inhibitor. As a growth promoter in cancerous cells, TGF-β fosters metastasis and invasiveness through stimulating *MDM2* overexpression, which in turn knocks off TP53 balance [49]. On the other hand, as a tumor suppressor, it discourages the growth of epithelial[50] and lymphoid cells by suppressing c-Myc and cyclin-dependent kinases (CDKs) while upregulating the expression of CDK inhibitors [51]. Epithelial cells with a sustained increase in MDM2 expression overpower the tumor inhibitory role of TGF-β[52] and allow transition from epithelial to mesenchymal cells through re-regulating Snail, vimentin, E-cadherin, and N-cadherin [53]. In breast cancer cells, elevated MDM2 levels were correlated with resistance against TGF-β1 treatment [33]. In case of a transient increase in MDM2 expression, no resistance to TGF- β anti-growth function was observed [54]. Thus, the inability of MDM2 to provoke resistance to TGF-β was related to the duration of exposure. Prolonged activation of *MDM2* in cells leads to the progression of cells from G1 to S phase circumventing TGF-β cell cycle arrest signals [54].

Regulation of steroid signaling including estrogen receptor (ERα and ERβ), androgen receptor (AR), and glucocorticoid receptor (GR) is paramount to the maintenance of cell physiological activities including cell growth and development process. The observations of aberrant regulation of GR in neuroblastoma [55], ER in breast cancer [56], and AR in prostate cancer [57] along with the elevated level of MDM2 in advanced stages evinced the strong correlation of MDM2 with steroid regulation. MDM2 in association with TP53 and ERα is shown to regulate ERα turnover in both estrogen-dependent and estrogen-independent manner [58, 59]. The transcriptional activity of ERα enhances multifold under the influence of high levels of MDM2 in the absence of the TP53 inside the cells [60]. The authors also showed that in a subset of breast cancer mutants, MDM2 activates the E2F1 pathway via phosphorylation of Rb.

The transcriptional activation of ER can be achieved by the interaction of ligand-bound ER or ligand free-ER to estrogen-responsive element (ERE) of estrogen-responsive target genes promoter (e.g., Presenilin*-2* (*pS2)*)[61]. In absence of the ligand, MDM2 followed by the proteasomal component Rpt6 are sequentially recruited to ERE of estrogen-responsive target genes. The complex promotes the swift degradation of the poly-ubiquitinated receptor with a fast turnover of 20 min, thus avoiding the accumulation of ER[58, 59]. Whereas ligand bound-ER binds to ERE of *pS2* with greater affinity than ligand-free receptor, which recruits histone modifiers e.g., histone methylation transferase (HMT) and histone acetylation transferase (HAT) along with coactivators including SRC-1 (steroid receptor coactivator-1) and polymerase II (Pol II) to initiate the process of transcription of estrogen-responsive genes [62]. Moreover, the turnover period extends to 45 min and prolonged engagement of the promoter by Pol II allows the transcription of estrogen-responsive genes [63]. Consistent with these findings, the elevation of MDM2 and exposure to estrogen stimulates the growth of ER-α positive breast cancer cell line (MCF-7) while conferring sensitivity to endocrine therapy [61]. Interestingly, in the presence of estrogen, ER-α protects TP53 from inhibition by MDM2, allowing TP53 to enhance the transcription of *MDM2* via MDM2/TP53 autoregulatory loop [64]. The *ligand-bound* ER can also upregulate MDM2 expression by interacting with promoters in the vicinity of the TP53 binding site. Hence, compounded effects of MDM2/TP53 loop and ER-α mediated MDM2 regulation in the presence of estrogen boost the level of MDM2 [65].

MDM2 regulates AR at various levels. MDM2 ubiquitinates AR to regulate AR levels crucial to maintaining normal cellular physiology [66]. MDM2-based regulation of AR involves the androgen-responsive elements (ARE) possessed by AR target genes. In a complex formed by the association of AR with HDAC-1 (histone deacetylase-1) and MDM2, MDM2 ubiquitinates the other two partners (HDAC-1 and AR) to reduce the transcription of AR. To achieve optimal ubiquitination, HDAC-1 deacetylation activity is required, suggesting the interplay between deacetylation and ubiquitination [67]. Co-activators of AR such as P300/CBP-associated factor (PCAF) and Tip60 (histone acetyltransferase (HAT) enzyme) are also potential targets of MDM2 [68]. The downregulation of AR is required for the maintenance of self-renewal capabilities in stem cells of prostate cancer [69].

The third genre of steroid receptors influenced by MDM2 expression levels is related to the family of glucocorticoid receptors (GR). Glucocorticoids bear the potential to provoke cell death or proliferation according to cell type and growth condition [70]. A group of genes containing GRE (Glucocorticoid Responsive Elements) is activated or repressed by GR[71]. In response to stress stimuli, a trimolecular complex containing TP53/MDM2/GR is formed where MDM2 suppresses the transcriptional activity of GR leading to apoptosis in mammary epithelial cells, vascular endothelial cells, and liver cells while enhancing survival in lymphocytes, lymphoma, and leukemia [72]. MDM2-mediated ubiquitination of GR takes place in the presence of TP53, i.e., the interaction of GR with TP53 requires MDM2 ligase activity. Thus, MDM2-mediated regulation of GR is highly dependent on TP53 levels in cells [73].

## MDM2 role in angiogenesis

The rapid growth and proliferation increase the nutrient requirement, hence cancerous tissues undergo the process of neovascularization to assure the continuous supply of nutrients. Among various angiogenic stimulators, vascular epithelial growth factor (VEGF) is a principal element stimulating angiogenesis in normal and pathological conditions [74]. The strict regulation and timely expression of *VEGF* are essential for the development of a normal vascular system and homeostasis [75]. In solid tumors, VEGF stimulates angiogenesis to promote cancer growth of tissues. A strong correlation between the high expression of VEGF and MDM2 has been found, implying the key role of MDM2 in VEGF-induced angiogenesis [76]. In a study on neuroblastoma cell line LA1-55 N, *VEGF* expression in *MDM2* deficient cells lessened considerably resulting in increased sensitivity to the chemotherapy [77]. The RING finger domain of MDM2 is involved in the stabilization of *VEGF* expression at the post-transcriptional level [76]. Under hypoxic conditions, MDM2 translocates from the nucleus to the cytoplasm and binds to the *VEGF* transcript to stabilize its expression [77]. By binding with hypoxia-inducible factor 1-α (HIF 1-α), which is induced in low oxygen tension, MDM2 upregulates the transcription of *VEGF* thus promoting neo-angiogenesis [78] (Fig. 1).

Studies have revealed that exposure to genotypic stress leads to dephosphorylation of MDM2 at S166 and S186, which is close to the nuclear localization signal (NLS) and nuclear export signal (NES), withdrawing MDM2 from the nucleus and forcing it to migrate to the cytoplasm [79]. Dephosphorylation of MDM2 at S166 is also evident in hypoxic conditions, thus setting the stage for redistribution of MDM2 in the cytoplasm and ultimate binding of *VEGF* mRNA to increase its stability [77].

MDM2 also stimulates angiogenesis by preventing the stimulation of anti-angiogenic factors. A recent study revealed the potential of MDM2 to prevent the stimulation of the anti-angiogenic factor, Von Hippel-Lindau (VHL*)*, by neddylation. The neddylation of VHL disables its interaction with TP53, leading to the inactivation of anti-angiogenesis process [80]. In renal cell carcinoma, VHL suppresses HIF thus inhibiting its interaction with MDM2 required for stabilizing the expression of *VEGF* [81].

## MDM2 promotes metastasis

Metastasis involves the migration of cancerous cells from their place of origin to another suitable site to spread cancer. The cellular intravasation depends on epidermal to mesenchymal transition (EMT) of cells, intravasation into blood, extravasation at the appropriate site, and eventually conversion from mesenchymal to epidermal (MET) cells to settle and establish a new population of tumorous cells at the new site [82]. During EMT, cancerous cells modify their identity by loss of epithelial properties and gain of mesenchymal characteristics [83]. The process of EMT enabling dissemination and invasion of the cells include the acquisition of mobility, invasiveness, and potential to disintegrate the complex network of extracellular matrix (ECM) [84, 85]. Although EMT possesses similarity in key cellular events, the critical details differ according to tissue nature and site [86].

**Fig. 3:**
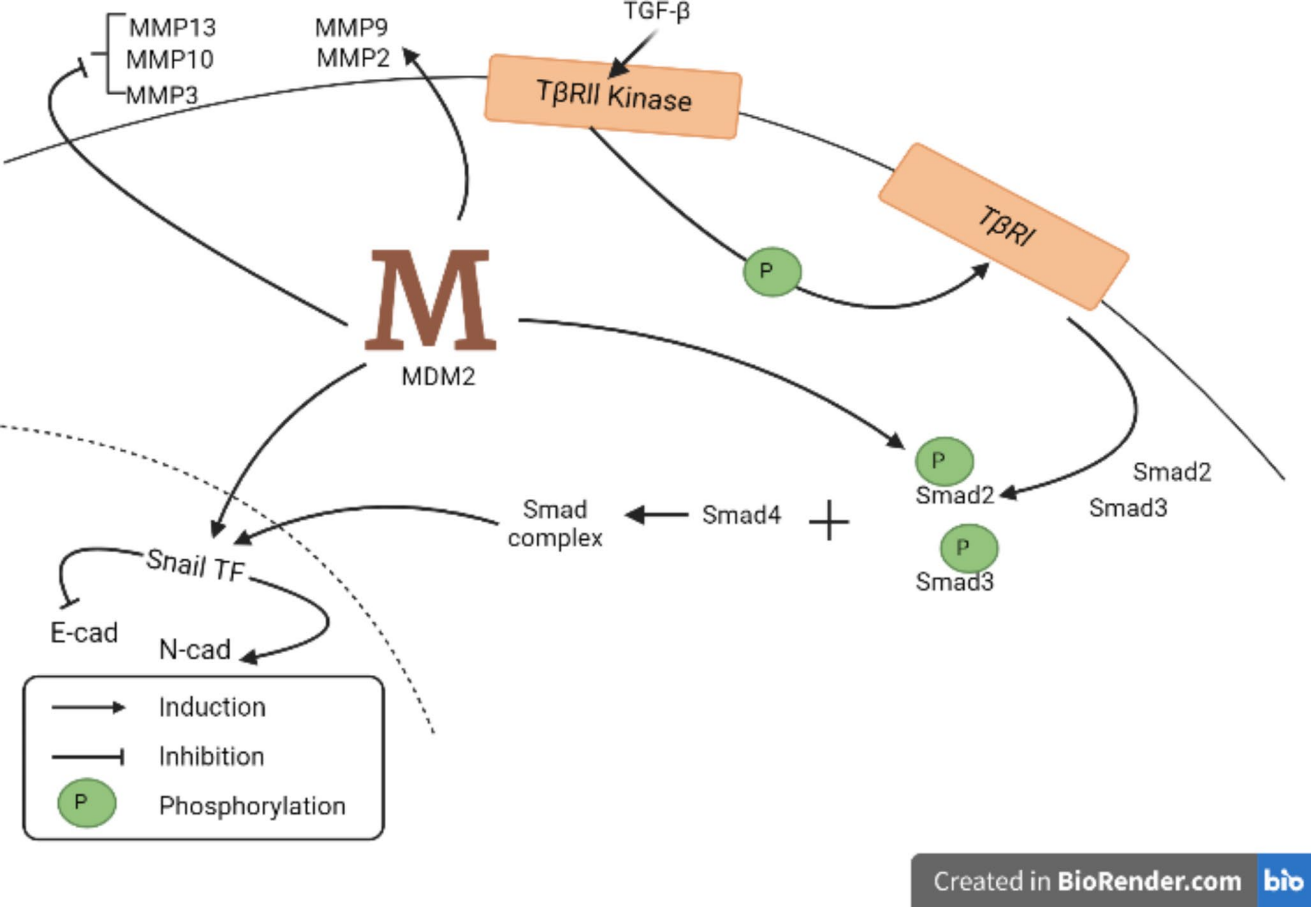
Role of MDM2 in metastasis. Upon TGF-β induction, TβRIIKinase phosphorylates TβRI which further phosphorylates Smad2 and Smad3. Phosphorylated Smad2 and Smad3 combine with Smad 4 to form smad complex which enters the nucleus to induce snail transcription factor (TF). Snail TF regulates the expression of cadherins. MDM2 possesses the ability to phosphorylate Smad2 and induce Snail TF, MMP-2 and MMP-9 while inhibiting MMP-3, MMP-10 and MMP-13 Abbreviation: MDM2, Murine Double Minute 2; TGF-β, Transforming Growth Factor-beta; TβRIIK, type II receptor kinases; TβRI, type I receptor; MMP, Matrix Metalloproteinases; TF, Transcription factor; Smad, Small mothers against decapentaplegic; E-cad, E cadherin; N-cad, N-cadherin

Several studies advocate the promotive role of MDM2 in metastasis [87–89]. MDM2 induces EMT-related cellular events through the regulation of multiple mediators as demonstrated in Fig. 3. In a study, silencing the expression of *MDM2* in breast cancer led to decreased vascularization in primary tumor tissue along with a significantly lower number of circulating cells [89]. Another study on hepatocellular carcinoma (HCC) identified the significant role of MDM2 inhibitor (SP141) in the repression of metastasis [90]. An *in-vitro* study conducted on a human ovarian cancer cell line (SKOV3) demonstrated the role of MDM2 in promoting EMT through inhibition of E-cadherin and activation of various growth-promoting transcription factors e.g., TGF-β/Smads and Snail/Slug [48, 87]. MDM2 also possesses the capability to activate the Smads (small mothers against decapentaplegic) pathway independent of TGF-β by direct phosphorylation of Smad-2 [87].

In ovarian malignancies, MDM2 facilitates cell motility and EMT through crosstalk of TGF-β-Smads pathway [87]. TGF-β activates the type II receptor (TβRII) kinases to phosphorylate type I receptor (TβRI) which further stimulates Smad2/3 by phosphorylation. The trimer molecule complex resulting from the union of activated Smad-2, -3, and − 4, after entering the nucleus regulates the expression of key mediators of metastasis [91]. To explore the role of MDM2 in modulating TGF-β-Smad pathway, exogenous MDM2 was introduced in SKOV3 cell line, resulting in the upregulation of transcription and translation of Snail and Slug transcription factors [87]. Similar observations that silencing *MDM2* in lung adenocarcinoma repressed the transcription of Snail and Slug induced by TGFβ1-Smad pathway were reported in another study [48]. Surprisingly, instead of E3 ligase activity, the N-terminal domain of MDM2 is essential for cancerous cells to undergo EMT and migrate [87].

MDM2 is stabilized by an MDM2 binding protein (MTBP) which is an important regulator of MDM2. MTBP when coupled to MDM2 discourages its self-ubiquitination ability, hence protecting its integrity and allowing the degradation of many target proteins [92]. MDM2 overexpression in MCF-7 cells led to a subsequent increase in the level of mesenchymal markers (vimentin, N-cadherin) whereas the expression of E-cadherin (an epithelial cell marker) significantly dropped indicating the transition of the cells [93]. Upon knocking down *MDM2* in MDA-MB-231 cells expressing mesenchymal markers, the cells acquired epithelial characteristics by expressing higher levels of E-cadherin while lowering vimentin and N-cadherin expression levels [93]. A study on invasive ductal breast carcinoma revealed the role of MDM2 in facilitating the invasion of malignant tumors by mediating the expression of matrix metalloproteinases *(*MMPs). MMPs are zinc-dependent endopeptidases that remodel ECM using their proteolytic abilities [94]. Knockdown studies on breast cancer cell lines establish the role of MDM2 in upregulating the expression while downregulating the expression of MMP-3, MMP-10 and MMP-13 [95, 96]. The upregulation of MMP*-*2 [89] and MMP-9 allows the breakdown of the extracellular matrix for tumor intrusion allowing the spread of cancer [36, 97].

## MDM2 enables metabolic reprogramming

**Fig. 4:**
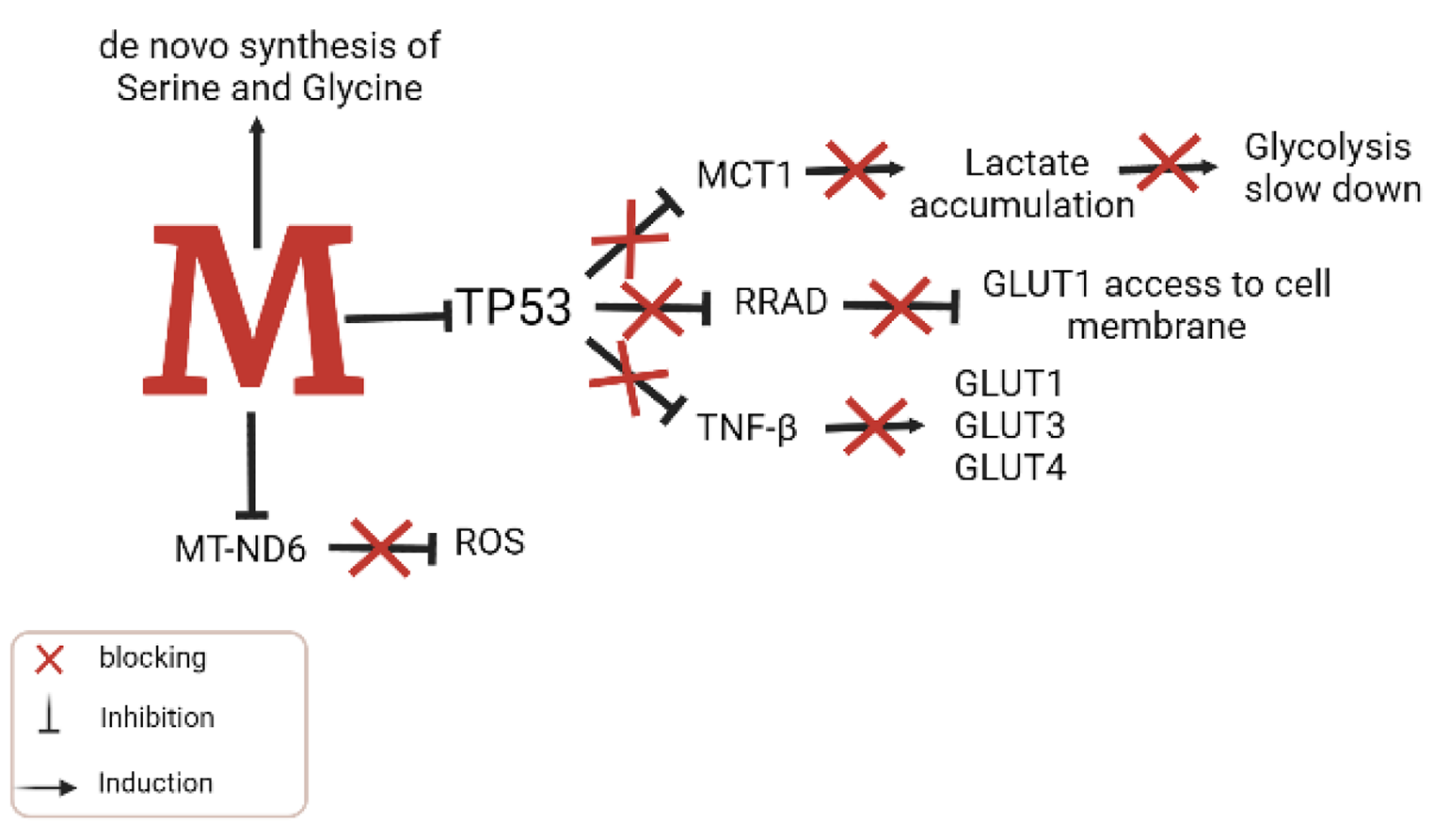
MDM2 in metabolic reprogramming. MDM2 regulates metabolism in TP53-dependent and -independent manner. Being the negative regulator of TP53, it manages to remove the inhibitory influence of TP53 from MCT1, RRAD and GLUT(1,3 and 4). In TP53 independent manner, it promotes the de-novo synthesis of Serine and Glycine. It also downregulates MT-ND6 to block its inhibition on production of ROS Abbreviation: MDM2, Murine Double Minute 2; TP53, Tumor Protein p53; MCT1, Monocarboxylate transporter 1; GLUT1, Glucose transporter 1; GLUT3, Glucose Transporter 3; GLUT4, Glucose Transporter 4; MT-ND6, Mitochondrially encoded NADH Dehydrogenase 6; ROS, Reactive Oxidative Species; RRAD, Ras-related glycolysis inhibitor and calcium channel regulator

MDM2-mediated metabolic reprogramming plays a fundamental role in the progression of cancer. Direct and indirect involvement of MDM2 in the metabolism of glucose, amino acids, and lactates signifies its potential to improve the survival of cancerous cells in an environment with scarce resource availability [98]. Figure 4 presents a brief overview of the diverse functioning of MDM2 in metabolic rewiring. Enhanced glycolysis is one of the peculiar adaptations of cancerous cells that enables cells to meet up increasing energy demands. TP53 negatively regulates the processes of glycolysis while MDM2 being the regulator of TP53 allows the continuation of cellular processes without any interference from TP53 [98]. TP53 obstructs the entrance of glucose in a cell by suppressing the transcription of *GLUT1*, *GLUT4*, or *GLUT3* through the inhibition of NF-κB [99]. TP53 also upregulates *RRAD* (Ras-related glycolysis inhibitor and calcium channel regulator) to hamper the access of GLUT1 to the plasma membrane [100]. Additionally, TP53 regulates lactate transportation by repressing the expression of MCT1 (monocarboxylic acid transporter 1) resulting in the accumulation of lactate in the cell that in turn slows down glycolysis [101]. In cancer cells, overexpression of MDM2 prevents the anti-glycolysis activities of TP53 by downregulation and degradation of TP53 [102].

MDM2 also initiates *de-novo* synthesis of serine and glycine when a cell faces a serine and glycine deficient environment [103]. In an experimental study, suppression of *MDM2* in cancerous cells exposed to serine and glycine-deficient medium eventually abated cell growth suggesting the significance of MDM2 in regulating serine and glycine metabolism [103]. Furthermore, MDM2 recruitment to chromatin allows the induction of transcription of genes involved in the synthesis, metabolism, and transport of serine and glycine amino acids [104]. In response to oxidative stress, mitochondrial localized MDM2 promotes the production of reactive oxygen species (ROS) by quashing the transcription of an NADH dehydrogenase (MT-ND6) that disrupts the respiration process [104]. The increased mitochondrial ROS production combined with decreased respiration is associated with enhanced metastatic potentials in cancerous cells [105]. In addition, MDM2 may impact super complex assemblage and complex I by sequestrating and degrading subunits of complex I i.e., NDUFS1 along with increased production of ROS and DNA damage [103].

## MDM2 suppresses immune response

Cancer cells gain the ability to evade immune checkpoints by secretion of molecules that bind to T-cells to inhibit their response. As the interaction of tumor cells with T-cells and subsequent inactivation of T-cells play a key role in the endurance of tumor cells. Hence, immune checkpoint inhibitors (ICI) have been viewed as potential therapeutic choices to hamper the success of tumors. However, the development of resistance in most patients receiving ICI [106] and hyper progressiveness[107] pose real challenges to its clinical application. Overexpression of MDM2 helps the immune evasion process through multiple channels. Detailed studies of hyper progressive disease (HPD) cells established a positive correlation with MDM2 expression in cells[108, 109]. In addition, high resistance in MDM2 over-expressive ovarian cancer cell lines has been observed against T-cell mediated death whereas silencing MDM2 results in enhanced sensitivity[110]. Moreover, in a TP53-independent manner, the expression of proinflammatory cytokine interleukin 6 (IL-6) decreased significantly in *MDM2* knockdown cell lines [110], suggesting an immunosuppressive role of MDM2 in part via IL-6 regulation.

Guo and colleagues previously reported that TP53 activation by an MDM2 inhibitor (Nutlin-3) led to the secretion of DAMPs (damage-associated molecular patterns) resulting in TP53-dependent immunogenic cell death [111]. In another recent study, it was demonstrated that MDM2 blockade triggers an immune response, which is further accentuated by inhibition of the PD-1/PD-L1 pathway[112]. The overexpression of PDL-1 (Programmed death-ligand 1) is narrated in multiple tumor classes and the binding of PDL-1 with PD-1 (receptor) of T cells inhibits T cells[113]. Thus, providing a rationale for co-treatment with MDM2 inhibitors and immune checkpoint-blocking antibodies in cancer patients with wild-type TP53. Although a correlation between the high expression of MDM2 and immunosuppressive activities of cancer cells has been established, the underlying mechanism is yet to elucidate.

## Resistance fostering by MDM2 against therapeutic agents

The hypothesis that MDM2 provokes anti-therapeutic resistance in human malignancies was initially validated through a study performed on epidermoid carcinoma where the MDM2-p53 regulatory loop contributed to the development of resistance against cisplatin[114]. Cisplatin-induced phosphorylation of TP53 inhibits TP53 resistance response meanwhile switching on an auto-regulatory loop that results in an increased level of MDM2 and non-phosphorylated TP53, thus instigating cells to resist therapeutics [115]. MDM2 also induces resistance against cisplatin by downregulating TP53 [116]. The elevated expression of *MDM2* renders resistance to doxorubicin by downregulating the expression of WT TP53. *In-vivo* study on breast cancer demonstrated that the cells transfected with MDM2 showed high resistance to doxorubicin. In addition, the level of MDM2 was higher in doxorubicin-resistant cells than in doxorubicin-sensitive cells [117].

Research showed that mere targeting of the TP53-MDM2 loop does not yield the desired outcomes as the presence of excessive MDMX suppresses TP53 transcription to regulate TP53 levels[118]. Additionally, tying MDMX with MDM2 heightens the enzymatic degradative activity of MDM2 for TP53 [119]. Hence, drugs targeting both MDM2 and MDMX might prove better therapeutic options to reactivate TP53 [120]. For instance, following treatment with Inulanolide A, a drug that hampers the binding of MDM2-MDMX, reduced proliferative and invasive potentials were observed in prostate cancer [119]. Another study on triple-negative breast cell lines and mice model validated the synergistic effect of MDM2-MDMX inhibitors with doxorubicin in restraining cell viability, fostering apoptosis or cell cycle arrest, and enhancing the chemosensitivity [121].

MDM2 limits the success of radiotherapeutic treatment by reducing the sensitivity of cancerous cells through the MDM2-TP53 loop and EMT pathway [122]. MDM2 inhibitors have been shown to boost the probability of success of radiotherapy [111,123]. Following the treatment of tumor cells with MDM2 inhibitor (MI-219), TP53 degradation declined and the sensitivity of cancerous tissue to radiation increased significantly. In another strategy to prevent MDM2 and TP53 interaction and subsequent degradation of TP53, adenovirus-mediated TP53 gene therapy was found to enhance the sensitivity of cells toward radiation [124]. A study on gossypol (a natural product extracted from cotton) revealed its anti-cancerous capabilities by targeting the MDM2-VEGF pathway. Gossypol not only disrupts the MDM2-mediated stabilization of VEGF mRNA but also induces MDM2 to undergo an auto-ubiquitination process thus inhibiting oncoprogression by targeting angiogenesis along with anti-apoptosis [125].

## Anti-MDM2 in clinical research

MDM2 is one of the most studied molecules due to its direct regulation of p53 which could be used for inducing apoptosis in cancerous cells. Various molecules have been synthesized to disrupt the MDM2-p53 regulatory loop and induce cell death. Nutlin 3a, an analog of low molecular weight cis-imidazoline, displaces MDM2 from p53 and binds itself to TP53 binding pocket of MDM2 thus freeing p53 to initiate a cellular response to genotypic damages [125]. Although Nutlin 3a proved highly efficient in killing cancerous cells during in-vitro trials but its low specificity hampered further clinical research. Other derivatives of Nutlin 3a including RG7112 (RO5045337) and RG7388 (RO5503781, Idasanutlin) were synthesized and subjected to clinical trials. RG7112 showed high specificity but low potency. Although it underwent clinical trial phase I to evaluate optimal dosage in solid and hematologic tumors but could not continue up to phase II and III. Among Nutlin derivatives, RG7388 is regarded as the most efficient one for its high specificity and potency to kill cancerous cells as RG7388 restricted the growth of SJSA1 human osteosarcoma xenograft tumors at quantity equivalent to one quarter of RG7112 [125]. In vivo study with CGM097 (an MDM2 inhibitor) and OTX015 (a Bromodomain and Extra-terminal domain (BET) inhibitor) showed the reactivation of p53 in neuroblastoma. Another MDM2 inhibitor molecule BI907828 has been found effective in xenograft models carrying patient-driven MDM2 rich dedifferentiated liposarcoma [125]. Milademetan (DS-3032) has been found safe in clinical trial 1 in the Japanese population and is now in process of further clinical evaluation [125]. Siremadlin (HDM2) was also found safe and capable of inhibiting MDM2 in solid malignancies and lymphomas [125].

MDMX positively regulates MDM2 while MDM2 through a negative feedback process downregulates MDMX. The interaction between MDM2 and MDMX is targeted through small molecule NSC207895 in hepatoblastoma which inhibited MDMX ability to upregulate MDM2. This in turn decreased the MDM2 level to an extent that its inhibitory effect on p53 diminished to cause effect and apoptosis took place [125]. SP-141 is another unique inhibitor that possesses the ability to induce autoubiquitination in MDM2 molecule thus its degradation. The studies on pancreatic cell lines and xenograft tumors in mice models validated the cytotoxic and regressive potentials of SP-141 [125].

MDM2 also plays a critical role in supporting the process of angiogenesis in tumors. Its interaction with HIF1-α is targeted through Serdemetan to weaken the stimulation of VEGFA. The effect can be further increased by co-inhibition of MDM2 and VEGFA resulting in low vascularization and slowing down the progression of tumors [125]. Gossypol inhibits the interaction of mVEGF and MDM2 thus destabilizing the mVEGF. As a result, the process of neovascularization gets impaired. Gossypol also regulates MDM2 by prompting its autoubiquitination capability [125].

The immunosuppressive potential of MDM2 is another major challenge in achieving clinical goals. A recent study evaluating the combination of APG-115 (an inhibitor for MDM2) with pembrolizumab (antibody targeting PD-1) showed a synergistic effect through the enhancement of immunity against tumors [125]. AMG-232 inhibition of MDM2 lowers the expression of IL-6 which consequently sensitizes MDM2 upregulated tumor cells to T cell-mediated death [125].

In conclusion, research over the past two decades has unveiled the complex picture of MDM2 as a regulator of multiple cellular processes. Beyond being a mere oncogenic protein, MDM2 has been established as a novel player controlling various aspects of cellular physiology. Taking together, the pivotal role of MDM2 in cancer development is of great significance for the development of therapeutic solutions.

## Conclusion

In conclusion, research conducted over the past two decades has revealed the intricate role of MDM2 in regulating multiple cellular processes. Although MDM2 promotes cancer growth through its stimulation of angiogenesis, metastasis, and metabolic reprogramming, its anti-apoptotic roles are important for the development of therapeutic solutions. The MDM2-TP53 autoregulatory loop, which has been extensively studied in the regulation of apoptosis, has sparked clinical research to find and develop anti-cancer therapies. By inhibiting TP53 through its E3 ligase ability, MDM2 enables cancerous cells to evade apoptotic signaling. MDM2-mediated regulation of cell cycle progression is achieved by activating and inhibiting various genes involved. Additionally, in metastasis, the expression of MDM2 regulates EMT and MET. MDM2 also contributes to the development of vascularization to support the growing nutritional demands of cancers. To enable the adaptation of cancerous cells to a stressful environment with limited resources, MDM2 introduces changes in metabolic pathways, including glycolysis, and stimulates the de novo production of amino acids such as glycine and serine through modulation of key processes. Overexpression of MDM2 is associated with poor prognosis and advanced stages of cancer, making it a promising target for the development of anti-cancer therapies.

## Data Availability

Not applicable.

## Abbreviations

AR: Androgen Receptor
ARE: Androgen Responsive Elements
CDK: Cyclin-dependent Kinase
DP1: Dimerization partner 1
E2F: Elongation factor 2
ECM: Extracellular matrix
EMT: Epidermal to Mesenchymal Transition
ER: Estrogen Receptor
ERE: Estrogen Responsive Elements
GR: Glucocorticoid Receptors
GRE: Glucocorticoid Responsive Elements
HAT: Histone Acetyltransferase
HCC: Hepatocellular Carcinoma
HDAC: Histone Deacetyl transferase
HDM2: Human Double Minute 2
HIF 1-α: Hypoxia-inducible factor-alpha
LAC: Lung Adenocarcinoma
MDM2: Murine Double Minute 2
MET: Mesenchymal to Epidermal Transition
MMP: Matrix Metalloproteinase
MTBP: MDM2 Binding Protein
NES: Nuclear Export Signal
NLS: Nuclear Localization Signal
PCAF: P300/CBP-associated factor
PI3K: Phospho-Inositol 3 Kinase
PolII: Polymerase II
PS2: Presenilin-2
RB: Retinoblastoma
ROS: Reactive Oxidative Species
RP: Ribosomal proteins
Smad: Small mothers against decapentaplegic
SNP: Single Nucleotide Polymorphism
Src: Proto-oncogene tyrosine-protein kinase
SV40: Simian Virus 40
TGF-β: Transforming growth factor-beta
TP53: Tumor Protein p53
TβRI: TGF-β Receptor Type I
TβRII: TGF-β Receptor Type II
VEGF: Vascular Epithelial Growth Factors
VHL: Von Hippel-Lindau
WRN: Werner Syndrome RecQ like helicase
